# Care pathways and factors associated with interhospital transfer to neurotrauma centers for patients with isolated moderate-to-severe traumatic brain injury: a population-based study from the Norwegian trauma registry

**DOI:** 10.1186/s13049-023-01097-7

**Published:** 2023-06-26

**Authors:** Mathias Cuevas-Østrem, Kjetil Thorsen, Torben Wisborg, Olav Røise, Eirik Helseth, Elisabeth Jeppesen

**Affiliations:** 1 Faculty of Health Sciences, University of Stavanger, Stavanger, Norway; 2 Department of Research, Norwegian Air Ambulance Foundation, Oslo, Norway; 3 Norwegian Trauma Registry, Division of Orthopaedic Surgery, Oslo University Hospital, Oslo, Norway; 4 INTEREST: Interprofessional Rural Research Team-Finnmark, Faculty of Health Sciences, University of Tromsø-the Arctic University of Norway, Hammerfest, Norway; 5 Norwegian National Advisory Unit on Trauma, Division of Emergencies and Critical Care, Oslo University Hospital, Oslo, Norway; 6 Hammerfest Hospital, Department of Anaesthesiology and Intensive Care, Finnmark Health Trust, Hammerfest, Norway; 7 Institute of Clinical Medicine, Faculty of Medicine, University of Oslo, Oslo, Norway; 8 Department of Neurosurgery, Oslo University Hospital, Oslo, Norway; 9 C/O Norwegian Air Ambulance Foundation, Postboks 414 Sentrum, Oslo, 0103 Norway

**Keywords:** Traumatic Brain Injury, Trauma system, Transfer, Interhospital, Geriatric

## Abstract

**Background:**

Systems ensuring continuity of care through the treatment chain improve outcomes for traumatic brain injury (TBI) patients. Non-neurosurgical acute care trauma hospitals are central in providing care continuity in current trauma systems, however, their role in TBI management is understudied. This study aimed to investigate characteristics and care pathways and identify factors associated with interhospital transfer to neurotrauma centers for patients with isolated moderate-to-severe TBI primarily admitted to acute care trauma hospitals.

**Methods:**

A population-based cohort study from the national Norwegian Trauma Registry (2015–2020) of adult patients (≥ 16 years) with isolated moderate-to-severe TBI (Abbreviated Injury Scale [AIS] Head ≥ 3, AIS Body < 3 and maximum 1 AIS Body = 2). Patient characteristics and care pathways were compared across transfer status strata. A generalized additive model was developed using purposeful selection to identify factors associated with transfer and how they affected transfer probability.

**Results:**

The study included 1735 patients admitted to acute care trauma hospitals, of whom 692 (40%) were transferred to neurotrauma centers. Transferred patients were younger (median 60 vs. 72 years, *P* < 0.001), more severely injured (median New Injury Severity Score [NISS]: 29 vs. 17, *P* < 0.001), and had lower admission Glasgow Coma Scale (GCS) scores (≤ 13: 55% vs. 27, *P* < 0.001). Increased transfer probability was significantly associated with reduced GCS scores, comorbidity in patients < 77 years, and increasing NISSs until the effect was inverted at higher scores. Decreased transfer probability was significantly associated with increasing age and comorbidity, and distance between the acute care trauma hospital and the nearest neurotrauma center, except for extreme NISSs.

**Conclusions:**

Acute care trauma hospitals managed a substantial burden of isolated moderate-to-severe TBI patients primarily and definitively, highlighting the importance of high-quality neurotrauma care in non-neurosurgical hospitals. The transfer probability declined with increasing age and comorbidity, suggesting that older patients were carefully selected for transfer to specialized care.

**Supplementary Information:**

The online version contains supplementary material available at 10.1186/s13049-023-01097-7.

## Introduction

Traumatic brain injury (TBI) is one of the leading causes of death and disability after trauma, resulting in approximately 2 million hospital admissions in Europe annually [1, 2]. The highest admission rates are seen in older patients, and as many countries face aging populations, this burden will likely increase [1, 2]. Systems ensuring continuity of care through the treatment chain improve outcomes for TBI patients [1, 3]. In current trauma systems, non-neurosurgical acute care trauma hospitals (ACTHs) are central in providing care continuity, but few studies have addressed their role in TBI management.

TBI patients primarily admitted to ACTHs may receive definitive care there or undergo interhospital transfer to a neurotrauma center (NTC) for access to neurosurgery or neurocritical care [4–7]. Patients who do not require neurosurgery or neurocritical care are recommended to receive definitive care at ACTHs. Furthermore, patients deemed ineligible for transfer due to unsalvageable injuries or have risk factors for a dismal prognosis despite interventions, such as very advanced age, significant comorbidity, or severe frailty, are also candidates for receiving definitive care at ACTHs [4, 8–15].

Previous studies have largely focused on patients admitted to NTCs [16, 17], so what characterizes patients presenting to ACTHs and their care pathways in a national system is poorly described. Moreover, the impact of advanced age and comorbidity on transfer decisions and treatment intensity is debated [18]. Studies of patients admitted to ACTHs have been limited to analyses of administrative databases [19], small sample sizes [14], narrow inclusion criteria [8], or data from a subset of hospitals in a national system [20]. More knowledge about the case-mix non-neurosurgical hospitals face and which patients they transfer to NTCs is important for further trauma system development [10].

We provide a population-based study from all ACTHs in a nationwide integrated trauma system. This provides a unique opportunity to investigate care for TBI patients outside NTCs from a setting that shares characteristics with trauma systems internationally, such as field triage tools and centralized neurosurgical services [7]. The Norwegian Trauma Registry’s (NTR) status as a national clinical quality registry warrants a law-regulated mandatory data delivery from all trauma-receiving hospitals by certified registrars. Understanding these patients’ care pathways and factors associated with transfer are important to evaluate how patients are identified for transfer, particularly regarding the older population. Consequently, this can inform targeted training, education, and system development to improve patient outcomes. The aim of this study was to describe characteristics and care pathways and identify factors associated with interhospital transfer to NTCs for patients with isolated moderate-to-severe TBI primarily admitted to non-neurosurgical ACTHs.

## Methods

### Study design and ethics

We extracted deidentified data from the NTR to conduct a national register-based study of adult cases with isolated moderate-to-severe TBI in Norway between January 1, 2015, and December 31, 2020, in line with the study protocol and the STROBE guidelines [21, 22]. Patients were compared across interhospital transfer status. Patients directly admitted to NTCs were included only for an overview of all care pathways and to calculate definitive care proportions. The NTR operates with a waiver of consent and all registered patients receive opt-out information. This study was approved by the Oslo University Hospital data protection officer (No. 19/16593). According to Norwegian legislation, approval from an ethics committee is not required for studies of deidentified registry data for health service research.

### Setting

Norway’s publicly funded healthcare system serves a population of 5.4 million people. A nationwide trauma system with uniform requirements for all prehospital services and all 38 hospitals has been implemented, including field triage criteria (Supplementary Fig. 1) [7, 23]. Patients with moderate-to-severe TBI receive 24/7 neurosurgical and neurocritical care services at all four regional referral trauma centers (TCs) and one ACTH (Stavanger University Hospital), jointly called NTCs in this study (Level I/II TCs [24]) (Supplementary Fig. 2). All 38 hospitals have 24/7 trauma team availability, emergency general surgery, and critical care and high-dependency units (i.e., CCUs/HDUs, intensive care, and postoperative care units).

### Data collection

The NTR is a mandatory clinical quality registry that has collected national data since 2015. Patients admitted to TCs and ACTHs who are (1) admitted through trauma team activation (TTA) or (2) admitted without TTA but found to have (a) penetrating injuries to the head, neck, torso, or extremities proximal to the knee or elbow, (b) head injury with Abbreviated Injury Scale (AIS) score ≥ 3, or (c) New Injury Severity Score (NISS) > 12, or (3) die at the scene of injury or during transportation to the hospital where prehospital management has been initiated are registered [25–27]. Registrars search electronic hospital databases and emergency admission protocols for patients not admitted through TTA who meet inclusion criteria. The estimated patient coverage is > 90% [26]. Data collection is based on the Utstein template, and injuries are coded according to the AIS manual 2005 (update 2008) by Association for the Advancement of Automotive Medicine certified nurse registrars [28, 29].

### Selection of participants

**Fig. 1:**
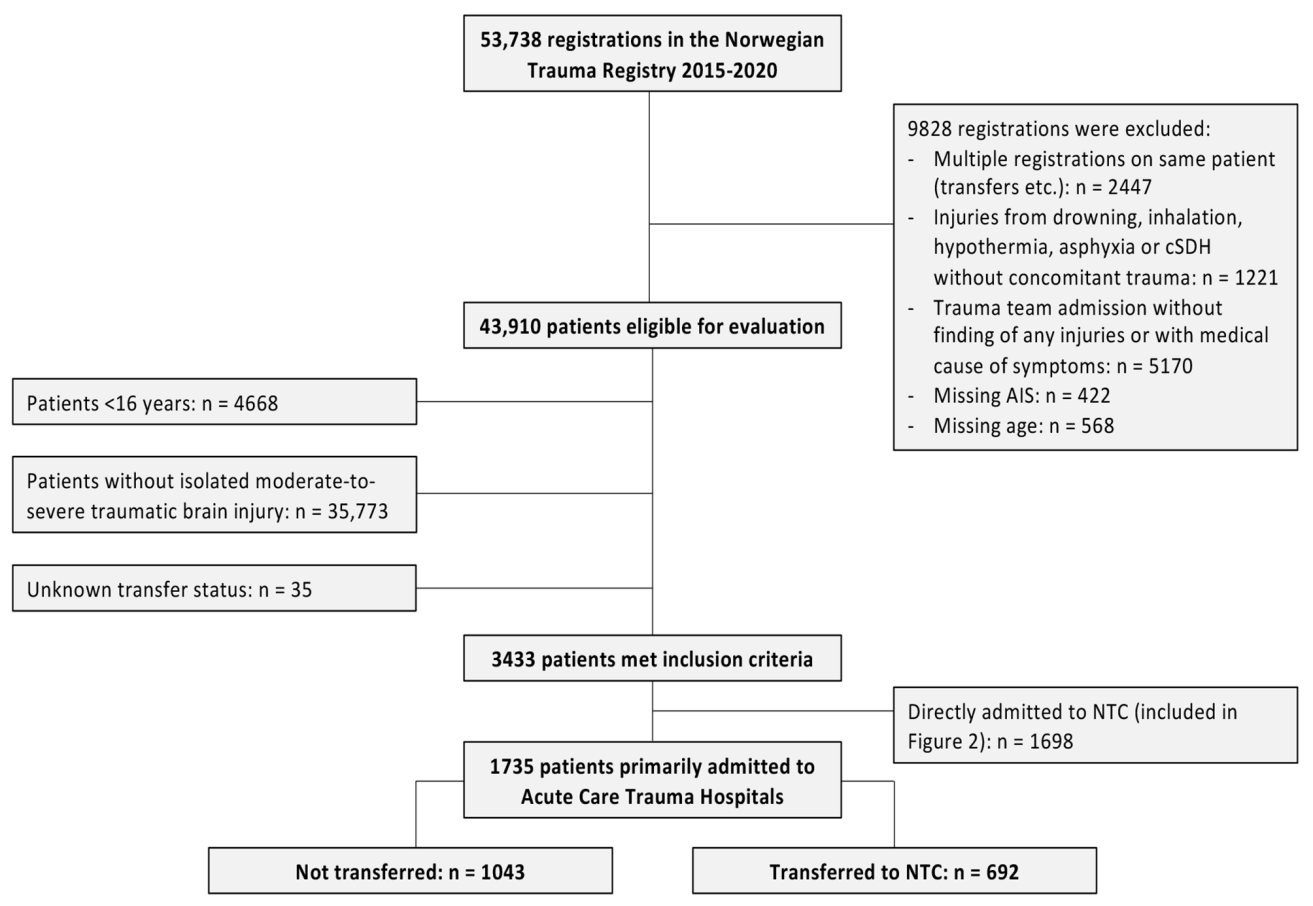
**Flowchart of the study population**. Abbreviations: AIS, Abbreviated Injury Scale; cSDH, Chronic subdural hematoma; NTC, Neurotrauma center

Patients primarily admitted to ACTHs aged 16 years or older with isolated moderate-to-severe TBI were included (Fig. 1). Isolated moderate-to-severe TBI, i.e., absence of significant extracranial injuries, was defined as (1) head injury with AIS scores of 3 to 6; (2) no extracranial AIS scores higher than 2; and (3) a maximum of one extracranial injury with an AIS score of 2. This definition was chosen to identify a population in which TBI would be the reason for considering a transfer to NTCs. It aligns with previous studies of isolated moderate-to-severe TBI and the Norwegian trauma guideline that recommends transfer of patients with injuries in three or more body regions, while allowing patients with extracranial injuries unlikely to affect transfer decision or outcome considerably to be included [7, 30, 31]. An AIS Head score of ≥ 3 reflects detectable intracranial pathology or cranial fractures on CT. Patients with chronic subdural hematoma without concomitant trauma were excluded. The study population was stratified by care pathways.

### Study variables

Patient characteristics were compared between transferred and nontransferred patients regarding demographics, injury characteristics, management, and mortality. Care pathways were reported as primary admission rates to ACTHs and NTCs, transfer rates from ACTHs to NTCs, and definitive care rates at ACTHs and NTCs. Patients primarily admitted to NTCs were included only in care pathway analyses (Fig. 1).

Variables extracted from the NTR are listed in Supplementary Table 1 with details regarding operationalizing. Information about head injury types was derived from AIS Head codes according to Supplementary Table 2. Patients with multiple different head injuries were registered in more than one category. NISS is calculated from the sum of the square of the three highest AIS codes irrespective of body regions, which, due to the study’s inclusion criteria, largely reflects the overall severity of head injuries [27]. The Glasgow Coma Scale (GCS) score was registered as the first GCS score upon admission unless the patient had undergone prehospital intubation, whereas the last GCS score before sedation was registered. GCS scores were categorized according to the Head Injury Severity Scale (HISS) [32]. Road distances from ACTHs to their corresponding NTCs according to the trauma plan were calculated using OpenStreetMap (www.openstreetmap.org/copyright – OpenStreetMap Foundation, Cambridge, United Kingdom) (Supplementary Table 3). The injury site’s municipality number was mapped to the 2017 urban-rural classification system Centrality Index of Norway and categorized as “major urban Norway”, “minor urban Norway” and “rural Norway” (categories 1 and 2, 3 and 4, and 5 and 6, respectively) [33].

### Statistical analyses

Continuous data with a nonnormal distribution are presented as medians with interquartile ranges (IQRs), and categorical data are reported as numbers and percentages. Baseline characteristics were compared using the Mann‒Whitney *U* test for continuous data and the χ² test for categorical variables. A *P* value *<* 0.05 (two-tailed) was considered statistically significant.

To identify factors significantly associated with interhospital transfer from ACTHs to NTCs, a generalized additive model (GAM) was developed according to the purposeful selection modeling strategy [34]. This procedure tests all relevant variables in univariable analysis for statistical significance before entering them into a multivariable model. Furthermore, the procedure tests for interaction and nonlinear terms and adds them if needed. Interactions reflect that the effects of each variable in the interaction term are not constant across all levels of the other variable. Age (continuous), sex, preinjury American Society of Anesthesiologists physical status (ASA-PS) [35], year of incident, injury site’s urban-rural classification [33], distance from ACTH to NTC (km, continuous), injury mechanism, GCS score on admission to ACTHs (HISS), and NISS (continuous) were considered important before analysis [11, 36, 37]. Because of missing data in some of the covariates (Table 1), multiple imputation was performed based on a missing at random assumption [38, 39]. For missing GCS scores on admission to ACTHs, next observation carried backward imputation was used instead of multiple imputation when GCS scores from an NTC were available. Following the purposeful selection procedure, the following interactions were deemed clinically relevant and evaluated in the model development: age*ASA-PS; age*NISS; age*ASA-PS*NISS; age*ACTH-NTC distance; NISS*ACTH-NTC distance; and age*GCS score. Assumptions about linearity were checked, and variables age and NISS were determined to be nonlinear. Estimates for the regression coefficients are presented as odds ratios (ORs) with 95% confidence intervals (CIs), and the effective degrees of freedom (edf) are given for nonlinear terms and visualized as contour plots. Statistical analyses were performed using SPSS v.27 (IBM Corp., Armonk, NY) and R statistical software (v.4.2.0; R Core Team, 2022) using the mgcv-package [40].

**Table 1:** Demographics, injury characteristics and outcome for adult patients with isolated moderate-to-severe traumatic brain injury primarily admitted to acute care trauma hospitals, by transfer status to a neurotrauma center

	All patients (n = 1735)	Not transferred (n = 1043)	Transferred (n = 692)	*P* value^a^	Missing
**Patient age**, median (IQR)	67 (49–80)	72 (53–84)	60 (42–71)	< 0.001	0.0%
**Sex**				< 0.001	0.0%
Female	563 (32.4)	387 (37.1)	176 (25.4)		
Male	1172 (67.6)	656 (62.9)	516 (74.6)		
**Preinjury ASA-PS**				0.015	3.7%
Normal health	572 (34.3)	334 (32.9)	238 (36.4)		
Mild systemic disease	641 (38.4)	392 (38.6)	249 (38.1)		
Severe systemic disease	423 (25.3)	276 (27.2)	147 (22.5)		
Severe systemic disease that is a constant threat to life	34 (2.0)	14 (1.4)	20 (3.1)		
**Mechanism of injury**				0.009	5.0%
Traffic-related	244 (14.8)	154 (15.3)	90 (14.0)		
Low-energy fall^b^	834 (50.6)	536 (53.2)	298 (46.4)		
High-energy fall	404 (24.5)	227 (22.5)	177 (27.6)		
Other	167 (10.1)	90 (8.9)	77 (12.0)		
**Injury site’s centrality class** ^c^				0.693	6.3%
Major urban Norway	483 (29.7)	291 (29.0)	192 (30.9)		
Minor urban Norway	938 (57.7)	587 (58.5)	351 (56.5)		
Rural Norway	204 (12.6)	126 (12.5)	78 (12.6)		
**Distance between ACTH and NTC** ^d^				0.376	0,0%
Kilometers, median (IQR)	103 (55–220)	103 (55–220)	123 (61–195)		
**NISS**, median (IQR)	22 (14–30)	17 (14–25)	29 (22–41)	< 0.001	0.0%
**Maximum AIS Head score**				< 0.001	0.0%
3	862 (49.7)	628 (60.2)	234 (33.8)		
4	402 (23.2)	249 (23.9)	153 (22.1)		
5-6^e^	471 (27.2)	166 (15.9)	305 (44.1)		
**GCS score on admission**				< 0.001	17.5%
14–15	933 (65.2)	746 (73.4)	187 (45.2)		
9–13	269 (18.8)	144 (14.2)	125 (30.2)		
<9	229 (15.6)	127 (12.5)	102 (24.6)		
**Type of head injury** ^f^					0.0%
Subdural hematoma	1120 (64.6)	635 (60.9)	485 (70.1)	< 0.001	
Skull fracture	818 (47.1)	391 (37.5)	427 (61.7)	< 0.001	
tSAH	742 (42.8)	367 (35.2)	375 (54.2)	< 0.001	
Contusion	731 (42.1)	349 (33.5)	382 (55.2)	< 0.001	
Epidural hematoma	191 (11.0)	53 (5.1)	138 (19.9)	< 0.001	
Brain stem	62 (3.6)	17 (1.6)	45 (6.5)	< 0.001	
**Highest level of in-hospital care** ^g^				< 0.001	1.1%
General ward	373 (21.7)	342 (33.3)	31 (4.5)		
CCU/HDU	1295 (75.5)	662 (64.5)	633 (91.7)		
Other	48 (2.8)	22 (2.1)	26 (3.8)		
**30-day mortality**	265 (15.7)	176 (17.4)	89 (13.1)	0.019	2.5%

Abbreviations: ACTH, Acute care trauma hospital; AIS, Abbreviated Injury Scale; ASA-PS, American Society of Anesthesiologists physical status; CCU/HDU, Critical care or high-dependency unit; GCS, Glasgow Coma Scale; IQR, Interquartile range; NISS, New Injury Severity Score; NTC, Neurotrauma Center; tSAH, Traumatic subarachnoid hemorrhage

Data reported as n (%) unless stated otherwise

^a^*P* values were derived from the Mann-Whitney *U* test for continuous data and chi-squared test for categorical data, testing the null hypothesis of no difference between strata

^b^ Low-energy fall is defined as a fall from standing or up to one meter

^c^ Centrality class according to Statistics Norway 2017 Centrality Index

^d^ Driving distance between the ACTH where the patient was primarily referred and the corresponding NTC according to the national trauma plan

^e^ A total of five patients had AIS Head scores of 6 and none were transferred

^f^ Type of head injury was derived from AIS codes. More than one type of head injury may be described per patient, including injuries with AIS Head scores < 3 for those who had at least one AIS Head score ≥ 3

^g^ Highest level of in-hospital care reported at the definitive care hospital level. Other includes emergency department, operating room, and other

## Results

### Study population characteristics, mortality, and care pathways

The study cohort included 1735 patients with a median age of 67 years (IQR 49–80); 68% were male, the median preinjury ASA-PS score was 2 (IQR 1–3), 35% had an admission GCS score ≤ 13, the median NISS was 22 (IQR 14–30), and 50% had an AIS Head score ≥ 4 (Fig. 1; Table 1). The unadjusted 30-day mortality was lowest in the transferred group (13.1% vs. 17.4%, *P* = 0.019) (Table 1).

**Fig. 2:**
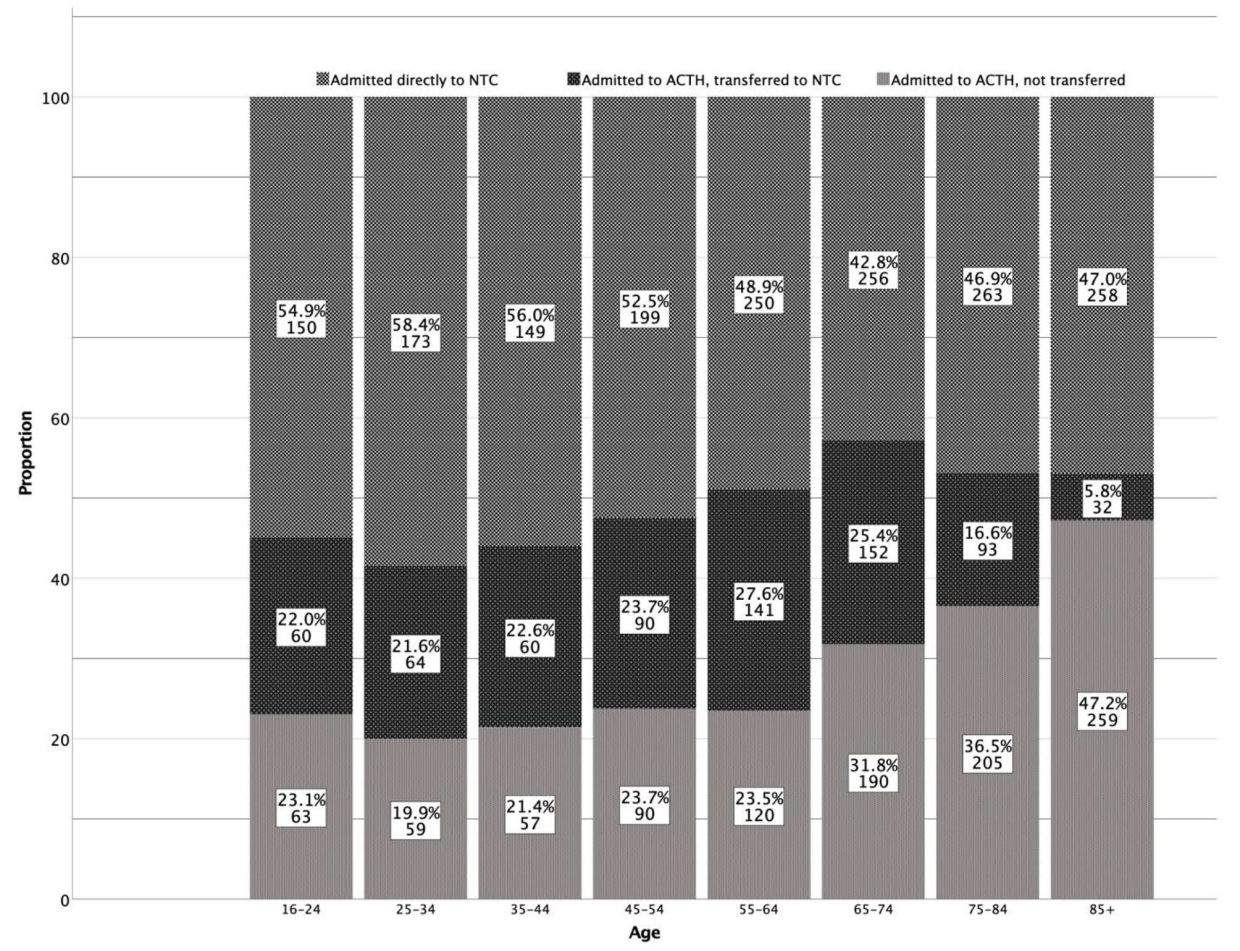
**Care pathways for patients with isolated moderate-to-severe TBI** Admission rates to ACTHs and NTCs as primary hospitals and interhospital transfer rates to NTCs, by age. Definitive care at an NTC is composed of patients directly admitted to an NTC and interhospital transfers. Primary admission to an ACTH is composed of patients not transferred and transferred from an ACTH. Abbreviations: ACTH, Acute care trauma hospital; NTC, Neurotrauma center

Forty percent (n = 692) of patients primarily admitted to ACTHs were transferred to NTCs (Figs. 1 and 2). Definitive care rates at NTCs were 76–80% for patients up to 65 years of age (Fig. 2). For those older than 65 years of age, this decreased with increasing age, resulting in 32–47% of patients receiving definitive care at ACTHs as a result of higher primary admission rates to ACTHs and decreasing interhospital transfer rates.

### Factors associated with interhospital transfer

Transferred patients were compared to nontransferred patients in univariate analysis (Table 1) and were younger (median 60 vs. 72 years, *P* < 0.001; proportion ≥ 65 years: 40% vs. 63%, *P* < 0.001), more often male (75% vs. 63%, *P* < 0.001), and had less comorbidity (preinjury ASA-PS ≥ 3: 26% vs. 29%, *P* = 0.015). Transferred patients were also more severely injured (median NISS: 29 vs. 17, *P* < 0.001; maximum AIS Head score ≥ 4: 66% vs. 40%, *P* < 0.001), had lower GCS scores at admission to ACTHs (GCS ≤ 13: 55% vs. 27%, *P* < 0.001), had higher frequencies of all head injury types (all *P* < 0.001), and had higher admission rates to CCUs/HDUs (92% vs. 65%, *P* < 0.001). The subgroups were injured in equally urban-rural parts of Norway and with similar distances between ACTHs and corresponding NTCs. Transferred patients were more severely injured across all age groups (Suppl. Figure 3).

**Fig. 3:**
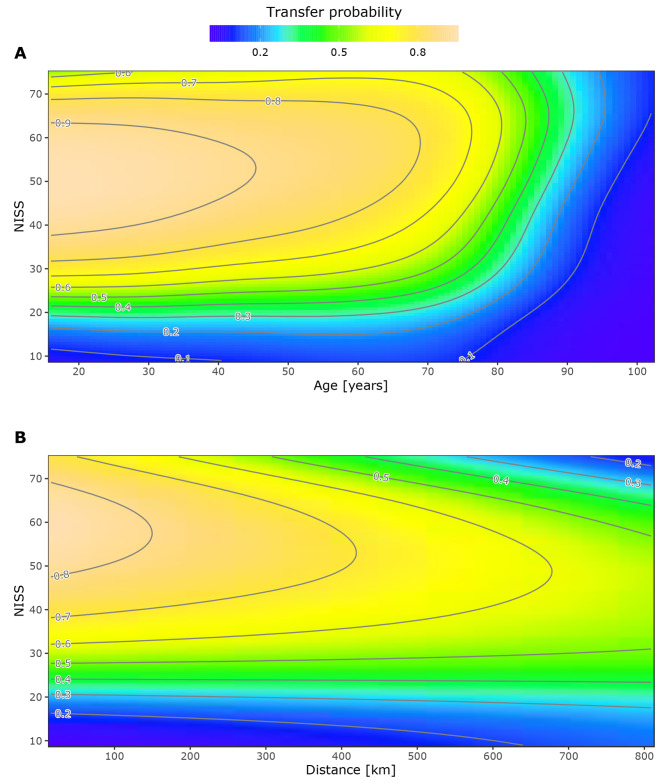
**Contour plots showing the estimated probability for interhospital transfer** as (**A**) NISS and patient age changes and (**B**) NISS and road distance between ACTHs and NTCs in kilometer changes, as a function of the full model. Other covariates are fixed at their median and mode values for continuous and categorical data, respectively. The distance between contour lines represents a 10% change in transfer probability

The final GAM (Table 2) identified factors significantly associated with interhospital transfer to NTCs and how they affected transfer probability. An increased transfer probability was associated with reduced GCS scores (GCS 9–13: OR 2.78 [95% CI 2.03–3.81], *P* < 0.001; GCS 3–8: 1.70 [95% CI 1.23–2.34], *P* = 0.001) and typically with increasing NISS. NISS interacted with age, and NISS’ effect on transfer probability showed an inverted U-shape for patients aged < 80 years, where the probability increased for NISSs up to 50–60 and decreased for higher NISSs (Fig. 3A). For patients with NISS > 20, a rapid decrease in transfer probability was observed from 70 to 80 years. For patients > 80 years, NISS had almost no impact on the transfer probability. A decreased transfer probability was associated with increasing age (Table 2), except for patients with preinjury ASA-PS 1–2 and extreme NISSs (< 15 or > 70) (Fig. 3A). Preinjury ASA-PS 3–4 was associated with an increased transfer probability for younger patients, but this effect rapidly decreased with age and was associated with a lower transfer probability compared to ASA-PS 1–2 patients from age 77 years (Suppl. Figures 4 and 5).

**Table 2:** Factors associated with interhospital transfer from acute care trauma hospitals to neurotrauma centers for patients with moderate-to-severe traumatic brain injury

	OR (95% CI)	edf	*P* value
GCS score			
14–15	1.00	N/A	
9–13	2.78 (2.03–3.81)	N/A	< 0.001
3–8	1.70 (1.23–2.34)	N/A	0.001
**Preinjury ASA-PS**			
1–2	1.00	N/A	
3–4	9.11 (1.66–49.94)	N/A	0.011
ASA 1–2*age	0.94 (0.90-0.997)	N/A	0.038
ASA 3–4*age	0.92 (0.87–0.97)	N/A	0.003
**Year of incident**	0.88 (0.82–0.94)	N/A	< 0.001
**s(age)**	N/A	2.92	< 0.001
**s(NISS)**	N/A	3.65	< 0.001
**ti(age, NISS)**	N/A	1.73	< 0.001
**ti(NISS, distance)**	N/A	0.88	0.008

Abbreviations: ASA-PS, American Society of Anesthesiologists physical status; edf, Effective degrees of freedom; GCS, Glasgow Coma Scale; N/A, Not applicable; NISS, New Injury Severity Score; s, smoothed term; ti, smoothed interaction term

NISS also interacted with the distance between the ACTH and the nearest NTC (Fig. 3B); for patients with NISSs greater than 30, the probability decreased with increasing distance; for NISSs between 20 and 30, the probability was roughly constant with increasing distance; and for patients with NISSs of 9–20, the probability increased slightly with increasing distance. A decreased transfer probability was also associated with the incident year (OR 0.87 [95% CI 0.82–0.94], p < 0.001), describing a decreased transfer probability throughout the study period.

## Discussion

In this population-based study from a national integrated trauma system, 40% of patients primarily admitted to ACTHs with isolated moderate-to-severe TBI were transferred to NTCs. Transferred patients were younger and more severely injured than nontransferred patients. An increased transfer probability was associated with factors reflecting the number and severity of head injuries; a reduced GCS score and an increased NISS, as well as comorbidities in patients aged < 77 years. However, for very high NISSs, the transfer probability declined. A decreased transfer probability was associated with increasing age and comorbidity in older patients, and distance between the ACTH and the nearest NTC, except in patients with extreme NISSs. These novel findings demonstrated that a substantial number of patients with isolated moderate-to-severe TBI were managed both primarily and definitively by ACTHs and that careful secondary triage was performed at admission to identify patients anticipated to benefit from specialized care.

The association between injury severity and transfer was as expected, as current guidelines emphasize GCS scores and radiological imaging results when evaluating TBI patients for transfer to specialized care [4, 7]. According to the study inclusion criteria, NISS largely reflected the number and severity of head injuries, and although NISS is a retrospectively calculated score, it is predominantly based on CT imaging, which is performed shortly after admission [41]. Interestingly, the transfer probability decreased for patients with NISSs > 50–60 and age < 80, reflecting a tipping point where injuries are so severe that transfer to advanced care in NTCs has likely been considered non-beneficial for some patients.

The Brain Trauma Foundation guidelines do not state an upper age limit for neurosurgical care [4]; however, studies have found advanced age to be associated with lower treatment intensity, and the incidence of emergency neurosurgery has been found to peak at age 75 [18, 42, 43]. Our study identified a rapid decrease in transfer probability at the same ages. Advanced age and comorbidities are known risk factors for poor prognoses [11, 44], and their association with reduced transfer probabilities is thus likely an expression of anticipated non-beneficence (Table 2; Fig. 3, Supplementary Fig. 4). Preinjury ASA-PS score 3–4 was, however, associated with an increased transfer probability for patients < 77 years, while it contributed to a decreased transfer probability for older patients (Supplementary Figs. 4 and 5). Comorbidities increase the risk of complicated clinical trajectories, which may be better managed at more resourceful hospitals. Thus, we believe this reflects a lower threshold for transfer in case of complications in patients expected to be able to benefit from specialized care.

Primary admission rates to ACTHs were highest for patients ≥ 65 years old (Fig. 2), which must be seen in light of prehospital triage tools’ limitations in detecting moderate-to-severe TBI for direct NTC transport in older patients [45]. Low-energy injury mechanisms are increasingly frequent with advanced age but are not well captured by the current triage guidelines (Supplementary Fig. 1), and caused 51% of injuries in the study population (Table 1). Studies have found that GCS scores are often higher for older adults than younger adults with similar anatomical injury severity [46]. The high proportion of patients with GCS scores of 14–15 at presentation to ACTHs in this study (64%, Table 1) indicates that this is an explanation for the observed increase in ACTH primary admission rates with age.

The interaction between NISS and the distance between ACTHs and NTCs was significantly associated with transfer (Fig. 3B). The increased transfer probability for patients with NISSs of 9–20 admitted to ACTHs far from the nearest NTC likely reflects proactivity regarding uncertain clinical development. The decreasing transfer probability with increasing distance for patients with NISSs > 30 likely reflects ‘the window of opportunity’ for performing successful neurosurgical interventions. Long transfer distances may cause a time to neurosurgery that exceeds this window even with the use of air ambulance transport. Interestingly, the distance between the ACTH and the nearest NTC was not significantly associated with transfer independently. Nor was the injury site’s centrality class, sex, or mechanism of injury, reflecting that these factors did not significantly affect transfer decisions.

Nontransferred patients had a higher 30-day mortality rate than transferred patients (Table 1), in line with previous studies [14]. This unadjusted mortality rate reflects the effect of NTC care, but also case-mix differences and the fact that the nontransferred subgroup encompassed patients treated with low intensity both due to nonsevere injuries and due to very severe injuries deemed unsalvageable or ineligible for specialized care (Table 1, Supplementary Fig. 3), as seen in other studies [13, 14, 18].

The incident year was negatively associated with transfer which suggests a decreased transfer probability throughout the study period. However, in the same period, the NTR matured, and increasing numbers of hospitals systematically searched for patients who met the registry inclusion criteria who had been admitted without TTA [26]. An increase in registrations of such patients from ACTHs over time would give the observed effect and is the most likely explanation for this finding. Therefore, it was necessary to adjust for this in the model. No changes in the trauma system have occurred that would cause a real decrease in transfer probability.

### Limitations

This study has limitations. First, the design is retrospective and observational, and we could only establish associations between various factors and probability for transfer, not cause-and-effect relationships. Second, information about factors that could have influenced transfer decisions beyond the confounders we adjusted for, including the use of antithrombotic medications, frailty, preinjury institutional living, pupil reactivity, GCS score deterioration (trends), neurological symptoms (e.g., lateralizing signs) or patient’s or relatives’ wishes, was not available from the NTR [7, 11, 12, 37, 44, 47]. This may have led to imprecise estimates, although most likely of minor impact because the included variables are those emphasized by current guidelines [4, 7]. Additionally, some of these factors with unavailable information are included in the national criteria for interhospital transfer as outlined in the national trauma plan (Supplementary Table 4), which we therefore could not use to evaluate transfer adequacy. Third, there is a risk that selection biases may have occurred from failure to identify patients at ACTHs for the NTR. The Scandinavian TBI guidelines’ recommendation of hospital admission for TBI patients with CT findings and the NTR’s registrar’s efforts to identify patients with AIS Head ≥3 likely counteracted this [26, 48]. In addition, the publicly funded health services likely reduced socioeconomically driven biases. Fourth, we used an AIS definition of moderate-to-severe TBI, which led to the inclusion of patients with mild TBI according to the HISS GCS classification (Table 1). Using a multidimensional measure of TBI severity has been advocated, e.g., combining AIS and GCS definitions. We chose to only use the AIS definition to include a population with a high degree of CT-diagnosed head injury reflecting real-world equipoise and practice for clinicians in ACTHs and to better capture older patients [46]. Fifth, the GAM contained six independent variables that cover a wide spectrum of potential patient cases. The dataset used to fit the model had poor coverage for atypical patients, e.g., patients with high preinjury ASA-PS (3–4) and ages below 50 years or patients with NISSs > 50. Thus, care should be taken to make inferences about atypical patients. Sixth, highly relevant information about neurosurgical interventions among patients who underwent transfer was unfortunately not available from the NTR but has been studied elsewhere [43]. Neurosurgical procedures are not performed outside NTCs in Norway. Finally, although the setting and demographics share important characteristics with other highly developed trauma systems, the generalizability may be limited by the mixed urban-rural geography and that the helicopter emergency service is integrated into the national health care system and frequently used for interhospital transfer.

## Conclusions

In conclusion, several of our findings suggest that patients with moderate-to-severe TBI admitted to ACTHs were managed with continuity of care within the trauma system: as much as 40% of patients admitted to ACTHs were transferred to NTCs; clinically available measures of severe injuries were associated with transfer; and some older adults seemed to be selected for transfer despite advanced age. ACTHs manage a large proportion of isolated moderate-to-severe TBI patients both primarily and definitively, which emphasizes the importance of trained staff in triage decisions and high-quality neurotrauma care in non-neurosurgical hospitals. Addressing the quality of neurotrauma care in ACTHs and whether factors other than those evaluated here are emphasized in these complex transfer decisions needs to be addressed in future research.

## Electronic supplementary material

Below is the link to the electronic supplementary material.

Supplementary Material 1

## Data Availability

The datasets used during the current study are available from the corresponding author upon reasonable request.

## List of Abbreviations

ACTH: Acute care trauma hospital
AIS: Abbreviated Injury Scale
ASA-PS: American Society of Anesthesiologists Physical Status
CCU/HDU: Critical care and high-dependency units
CI: Confidence Intervals
edf: Effective degrees of freedom
GAM: Generalized Additive Model
GCS: Glasgow Coma Scale
HISS: Head Injury Severity Scale
IQR: Interquartile Range
NISS: New Injury Severity Score
NTC: Neurotrauma Center
NTR: Norwegian Trauma Registry
OR: Odds Ratio
TBI: Traumatic Brain Injury
TC: Trauma Center
TTA: Trauma Team Activation
