# Supplementary material for: Care pathways and factors associated with interhospital transfer to neurotrauma centers for patients with isolated moderate-to-severe traumatic brain injury: a population-based study from the Norwegian trauma registry
Source: Scand J Trauma Resusc Emerg Med. 2023 Jun 26;31:34. doi: 10.1186/s13049-023-01097-7 (PMC10294301; doi:10.1186/s13049-023-01097-7)
Supplement: Supplementary file 1 — Supplementary Material 1 [file 13049_2023_1097_MOESM1_ESM.pdf]

Supplemental Online Content

To *Care pathways and factors associated with interhospital transfer to neurotrauma centers for patients with isolated moderate-to-severe traumatic brain injury: a population-based study from the Norwegian Trauma Registry*

List of contents:

- Suppl. Table 1: Variables extracted from the NTR.
- Suppl. Table 2: Categorization of AIS Head codes by pathoanatomic injury types.
- Suppl. Table 3: Road distances between ACTHs and corresponding NTCs.
- Suppl. Table 4: Transfer criteria (The Norwegian Trauma Plan)

- Suppl. Figure 1: Norwegian trauma field triage guidelines.
- Suppl. Figure 2: Overview of population density and neurotrauma centers and acute care trauma hospitals in Norway.
- Suppl. Figure 3: Age-stratified New Injury Severity Score (NISS) distributions for transferred and nontransferred patients.
- Suppl. Figure 4: Transfer probability by age and ASA-PS.
- Suppl. Figure 5: Transfer probability by age, NISS, and ASA-PS 3-4.

Suppl. Table 1: Variables extracted from the Norwegian Trauma Registry with details regarding operationalizing.

Variable name	NTR categories	Category operationalization
**Age**	continuous	N/A
**Sex**	Male	N/A
	Female	N/A
**Preinjury ASA-PS**	1-4 (no patients with >4)	Full and dichotomized (1+2, 3+4)
**Injury site municipality number**	N/A	N/A
**Mechanism of injury**	1 = Traffic: motor vehicle injury	1-5: Transport-related
	2 = Traffic: motorcycle injury	9: Low-energy fall
	3 = Traffic: bicycle injury	10: High-energy fall
	4 = Traffic: pedestrian	6, 7, 8, 11, 99: Other
	5 = Traffic: other	999 and missing: Missing
	6 = Shot by handgun, shotgun, rifle, other firearm of any dimension	
	7 = Stabbed by knife, sword, dagger, other pointed or sharp object	
	8 = Struck or hit by blunt object	
	9 = Low-energy fall	
	10 = High-energy fall	
	11 = Blast injuries	
	99 = Other	
	999 = Unknown	
**Emergency Department GCS**	3-15	N/A
**Highest level of in-hospital care**	1 = Emergency Department	2 = General Ward (no change)
	2 = General Ward	4 and 5 = CCU/HDU
	3 = Operation Theatre	1, 3, 6 = Other
	4 = High Dependency Unit (HDU)	
	5 = Critical Care Unit (CDU)	
	6 = Unknown	
**Abbreviated Injury Scale**	N/A	N/A
**New Injury Severity Score**	N/A	N/A
**30-day mortality**	N/A	N/A

Abbreviations: ASA-PS, American Society of Anesthesiologists physical status; GCS, Glasgow Coma Scale; N/A, Not applicable.

Suppl. Table 2: Categorization of Abbreviated Injury Scale Head codes to pathoanatomic injury types.

Pathoanatomic injury group	Injury category according to AIS manual	Injury codes	Severity code
**Skull fracture**			
	Skull fractures NFS	150000	2
	Base (basilar) fractures	1502xx	.3-.4
	Vault fractures	1504xx	.2-.4
**Vascular injuries**			
	Arterial, sinus, and venous injuries	12xxxx	.3-.6
**Nerve injuries**			
	Cranial nerve injuries	13xxxx	.2-.3
**Epidural hematoma**			
	Cerebellum epidural hematoma	140414; 140416; 140418; 140422	.2-.5
	Cerebrum epidural hematoma	140630; 140631; 140632; 140634; 140636	.2-.5
**Subdural hematoma**			
	Cerebellum subdural hematoma	140438; 140440; 140442; 140446	.2-.5
	Cerebral subdural hematoma	14065x	.3-.5
**Traumatic SAH**			
	Cerebellum tSAH	140466	2
	Cerebrum tSAH	140693; 140694; 140695	.2-.3
**Other extra-axial hemorrhage**			
	Cerebellum hematoma NFS	140410	3
	Cerebrum hematoma NFS	140629	3
	Cerebellum subpial hemorrhage	140470	2
	Cerebrum subpial hemorrhage	140696; 140697; 140698	.2-.3
**Brain stem**			
	Brain stem NFS	140299	5
	Brain stem compression, contusion, infarction	14020x	5

	Brain stem hemorrhage, laceration, massive destruction, penetrating, transection	14021x	.5-.6
**Brain contusion**			
	Cerebellum contusion	14040x	.2-5
	Cerebrum contusion	14060x; 14061x; 140620; 140621; 140622; 140624; 140626	.2-.5
	Intracerebellar hematoma	140426; 140428; 140430; 140434	.2-.5
	Intracerebral hematoma	140638; 140639; 14064x	.2-.5
**DAI**			
	Cerebrum	140628; 140625; 140627	.4-.5
	Concussive injuries; DAI	161007; 161008; 161011; 161012; 161013	.4-.5
**Penetrating**			
	Penetrating injury to skull	1160xx	.3-.5
	Cerebellum penetrating	140478; 140477; 140476	.3-.5
	Cerebrum penetrating	140690; 140691; 140692	.3-.5
**Other**			
	Crush injury	113000	6
	Cerebellum NFS	140499	3
	Cerebellum laceration	140474; 140473; 140472	.3-.4
	Cerebellum brain swelling/edema NFS	140450	3
	Cerebellum infarction (due to traumatic vascular occlusion)	140458	3
	Cerebellum ischemic brain damage direcetly related to head trauma	140462	3

	Cerebrum NFS	140699	3
	Cerebrum laceration	140688; 140687; 140686	.3-.4
	Cerebrum brain swelling NFS	140660; 140662; 140664; 140666	.3-.5
	Cerebrum brain edema NFS	140668; 140670; 140672; 140674	.3-.5
	Cerebrum infarction	140676	3
	Cerebrum intraventricular hemorrhage	140678; 140675; 140677	.2-.4
	Cerebrum ischemic brain damage directly related to trauma	140680; 140681; 140683	.3-.5
	Pneumocephalus directly related to head trauma	140682	3
	Pituitary injury	140799	3
	Concussive injuries	161000; 161001; 161002; 161003; 161004; 161005; 161006	.1-.3
	Scalp injuries	110099; 110202; 11040x; 11060x; 11080x	.1-.3
**.9-injuries not included**			
	Injuries to the head NFS	100099	9
	Died of head injury without further substantiation of injuries or no autopsy confirmation of specific injuries	100999	9
	Trauma-associated findings not related either to intervention or to anatomically-described head injury NFS	140689	9
	Cerebrum hypoxic or ischemic brain damage secondary to systemic hypoxemia, hypotension or shock not directly related to head trauma	14070x	9

Based on AIS 2005, update 2008
Injury codes including x denote all numbers with that prefix. Severity codes given as ranges reflect all severity codes possible for that injury category.
NFS: Not Further Specified.

Suppl. Table 3: Overview of road distances and drive times between acute care trauma hospitals and corresponding neurotrauma centers in Norway.

Neurotrauma center (NTC)	Acute care trauma hospital	Distance to regional NTC (km)*	Drive time (hh:mm)*
Haukeland University Hospital	Førde	179	03:16
Haukeland University Hospital	Haugesund	139	03:13
Haukeland University Hospital	Odda	136	02:41
Haukeland University Hospital	Stord	84	02:25
Haukeland University Hospital	Voss	103	01:31
St. Olav University Hospital	Kristiansund	197	03:21
St. Olav University Hospital	Levanger	80	01:09
St. Olav University Hospital	Molde	220	03:40
St. Olav University Hospital	Namsos	195	02:52
St. Olav University Hospital	Volda	347	06:39
St. Olav University Hospital	Ålesund	290	05:26
Stavanger University Hospital	N/A	N/A	N/A
UNN Tromsø	Bodø	538	07:51
UNN Tromsø	Hammerfest	439	08:46
UNN Tromsø	Harstad	303	04:09
UNN Tromsø	Kirkenes	809	10:43
UNN Tromsø	Lofoten	497	06:43
UNN Tromsø	Mo i Rana	658	09:11
UNN Tromsø	Narvik	235	03:15
UNN Tromsø	Sandnessjøen	767	10:49
UNN Tromsø	Vesterålen	409	05:34
Ullevål University Hospital	Ahus	18	00:22
Ullevål University Hospital	Arendal	258	02:59
Ullevål University Hospital	Bærum	16	00:21
Ullevål University Hospital	Drammen	43	00:43
Ullevål University Hospital	Flekkefjord	426	05:11
Ullevål University Hospital	Fredrikstad, Kalnes	90	01:09
Ullevål University Hospital	Gjøvik	123	01:54
Ullevål University Hospital	Hamar	129	01:28
Ullevål University Hospital	Kongsvinger	94	01:20
Ullevål University Hospital	Kristiansand	321	03:41
Ullevål University Hospital	Lillehammer	184	02:08
Ullevål University Hospital	Ringerike	55	01:00
Ullevål University Hospital	Telemark, Skien	135	02:03
Ullevål University Hospital	Tynset	326	04:04
Ullevål University Hospital	Tønsberg	103	01:19

*Distance and drive time estimates according to openstreetmap.org

Abbreviations: NTC, Neurotrauma center; UNN, University Hospital of Northern Norway.

Suppl. Table 4: Overview of selected transfer criteria for injuries to the head and CNS, according to the Norwegian Trauma Plan.

- Penetrating injury/open fracture
- Depressed fracture
- Lateralizing signs
- GCS deterioration
- GCS <14 with CT findings
- Spinal injury or unstable spinal/neck fracture

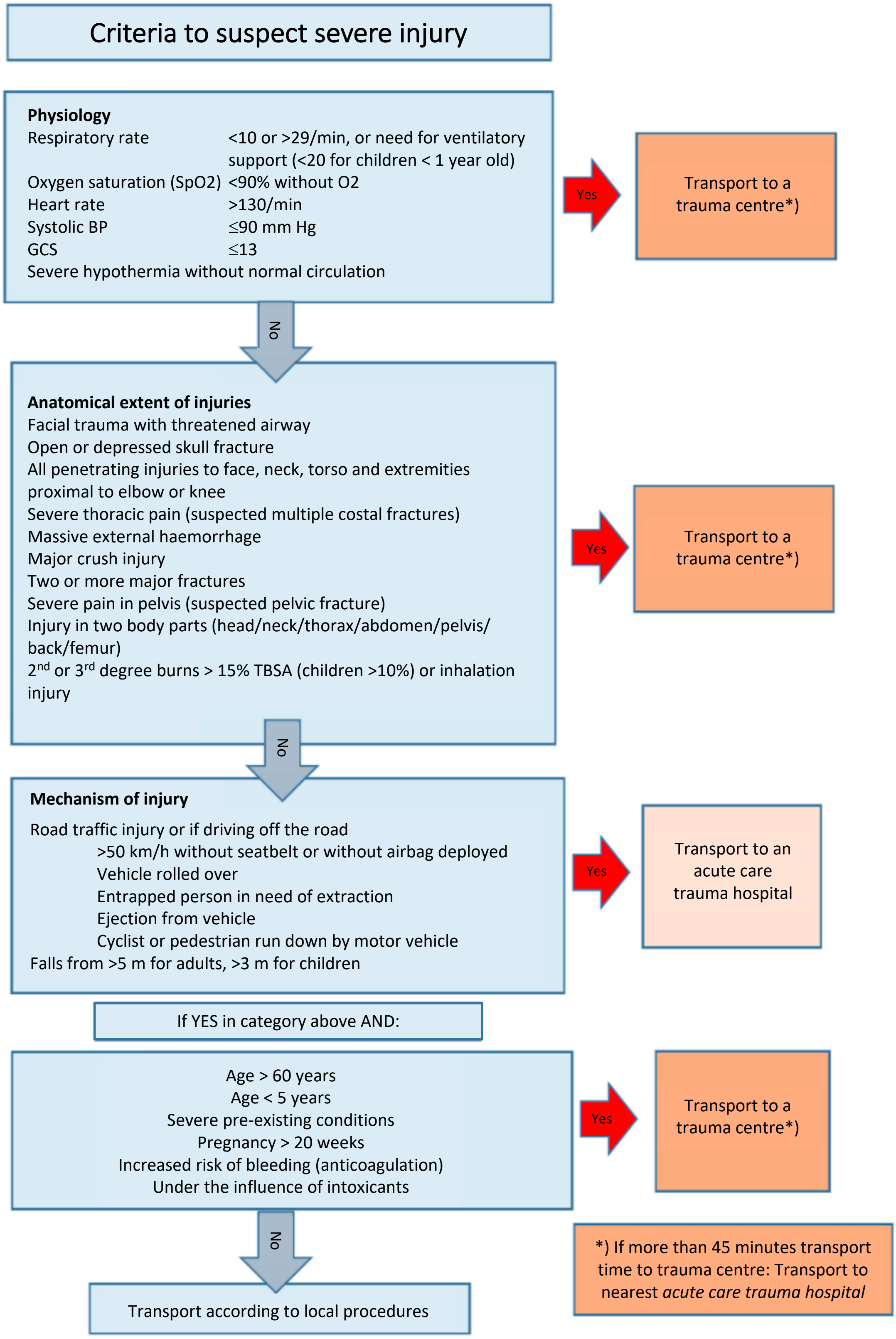

Suppl. Figure 1: The Norwegian trauma field triage criteria.

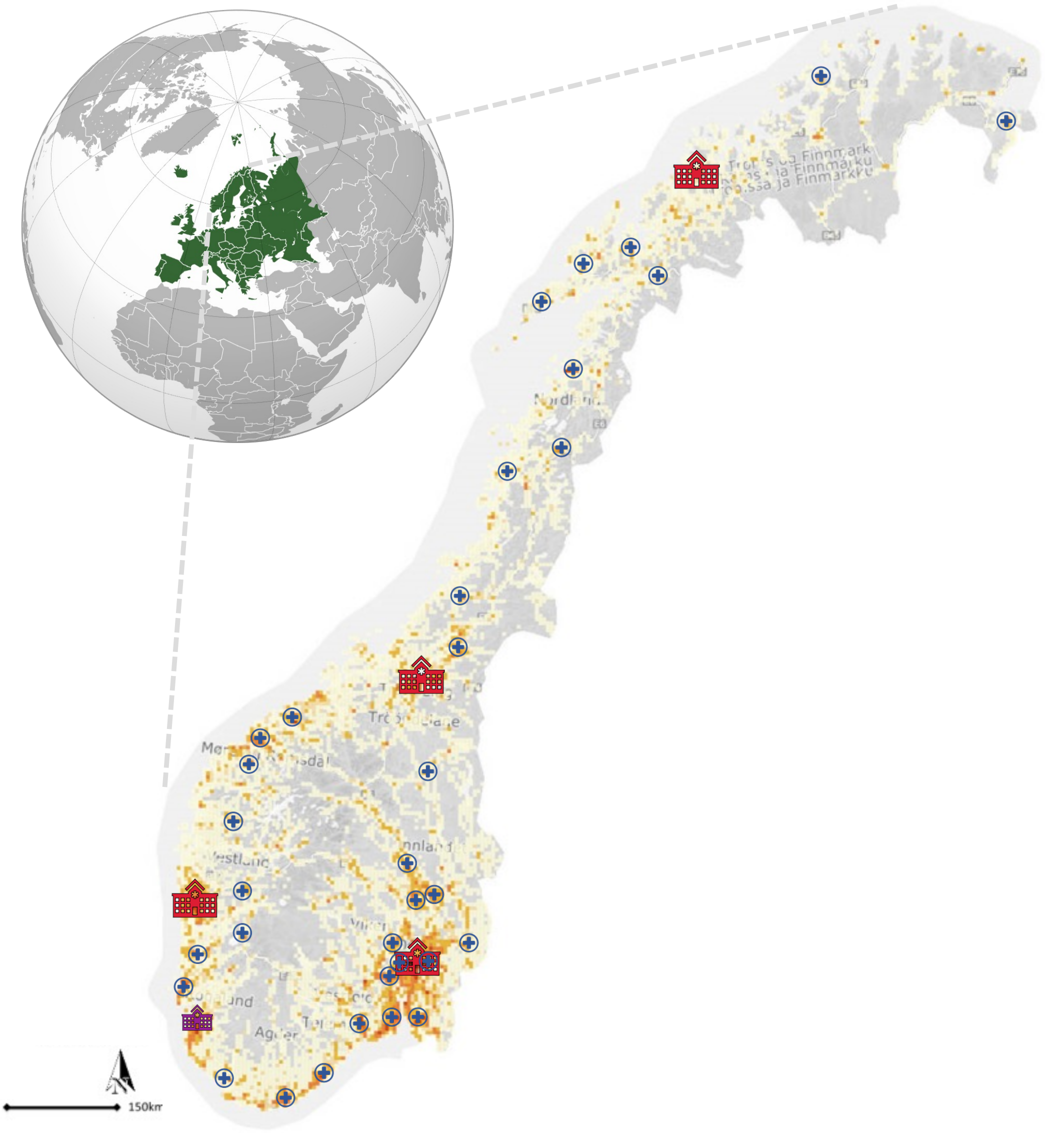

Suppl. Figure 2: Overview of population density and neurotrauma centers and acute care trauma hospitals in Norway.

A population density map of Norway with locations of neurotrauma centers (red for regional trauma centers and violet for the one acute care trauma hospital with neurotrauma services) and acute care trauma hospitals (blue) superimposed. Colors from light yellow to orange represent increasing population density. Left: Norway's location in Europe. *Public domain content from Wikimedia Commons and Statistics Norway.*

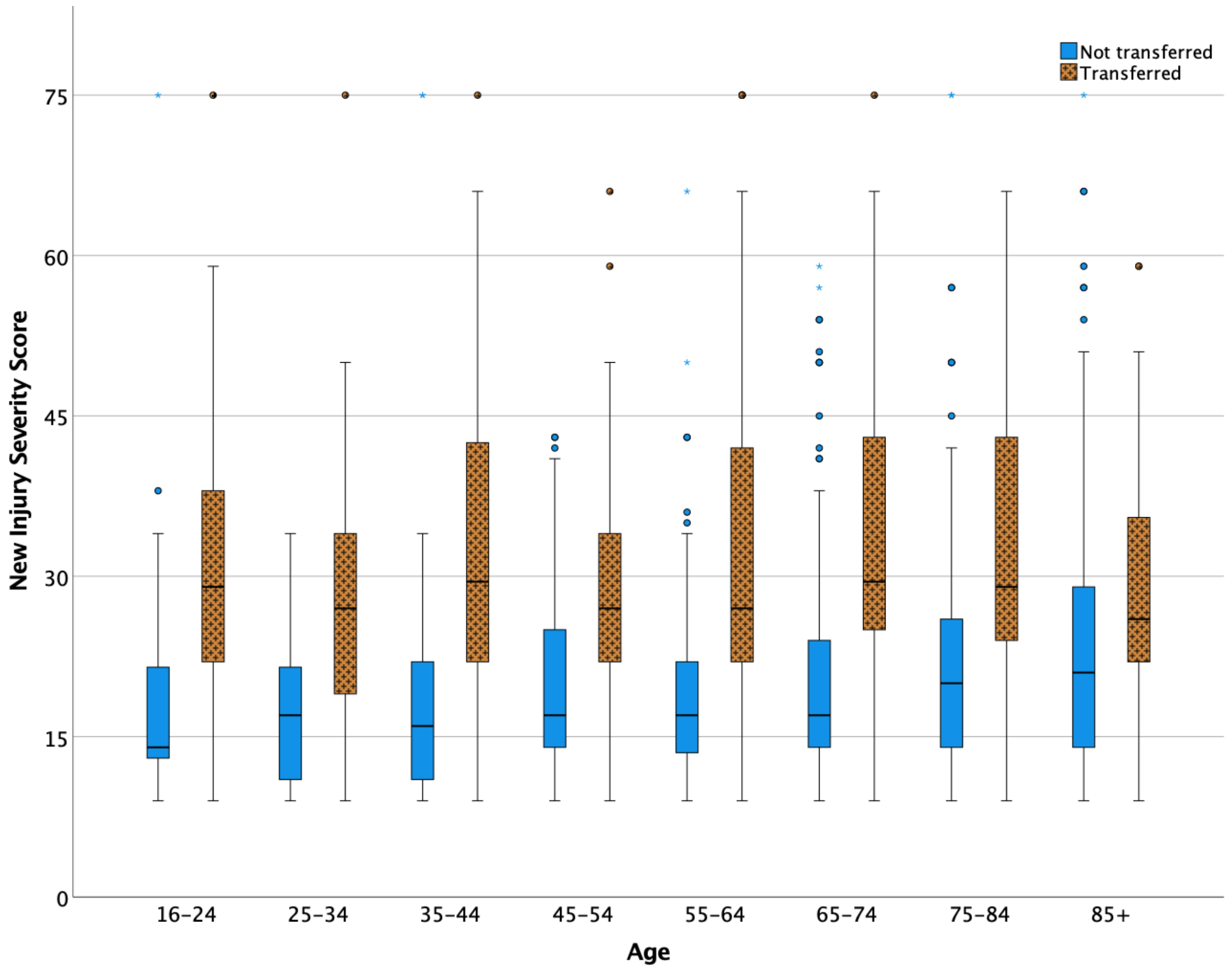

Suppl. Figure 3: Age-stratified New Injury Severity Score (NISS) distributions for transferred and nontransferred patients.
New Injury Severity Score (NISS) distributions for patients with isolated moderate-to-severe traumatic brain injury primarily admitted to acute care trauma hospitals, comparing nontransferred to transferred patients within age groups. The lower NISS range at 9 reflects the study inclusion criteria. Circles symbolize outliers and asterisks extreme outliers.

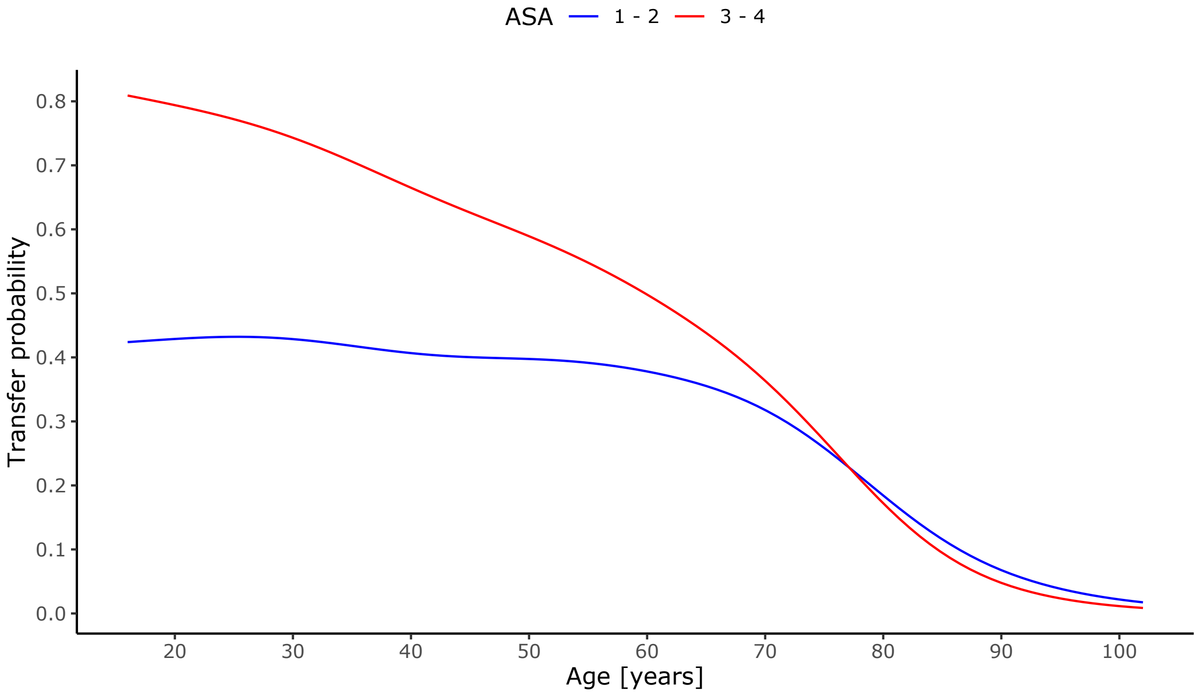

Suppl. Figure 4: Transfer probability by age and preinjury ASA-PS.
The probability of interhospital transfer for patients with isolated moderate-to-severe traumatic brain injury, comparing patients with preinjury American Society of Anesthesiologists physical status scores (ASA-PS) 1-2 with 3-4.

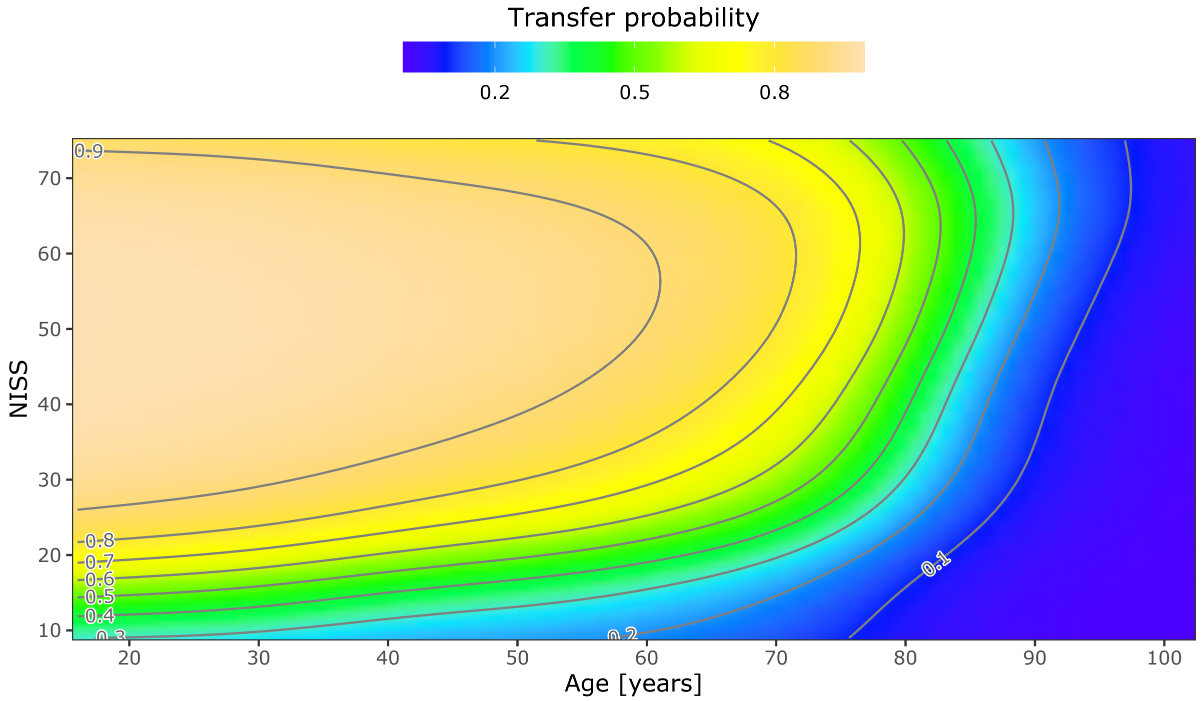

Suppl. Figure 5: Transfer probability by age, NISS, and preinjury ASA-PS 3-4. Contour plot showing the estimated probability for interhospital transfer as NISS and patient age changes for patients with preinjury comorbidity according to the American Society of Anesthesiologists physical status score (ASA-PS) 3-4, as a function of the full generalized additive model. Other covariates are fixed at their median and mode values for continuous and categorical data, respectively. For comparison with ASA-PS 1-2 patients, see figure 5A.
